# The Impact of E-Cigarettes on Oral Health—A Narrative Review

**DOI:** 10.3390/dj12120404

**Published:** 2024-12-10

**Authors:** Dominika Cichońska, Aida Kusiak, Maciej L. Goniewicz

**Affiliations:** 1 Department of Periodontology and Oral Mucosa Diseases, Medical University of Gdańsk, 80-208 Gdańsk, Poland; akusiak@gumed.edu.pl; 2 Department of Health Behavior, Roswell Park Comprehensive Cancer Center, Elm and Carlton Streets, Buffalo, NY 14203, USA

**Keywords:** e-cigarettes, oral health, caries, periodontitis, oral mucosa, oral microbiome, saliva

## Abstract

**Background/Objectives**: Electronic cigarettes (e-cigarettes) are commonly used by former smokers as an alternative product to conventional cigarettes and also by young adults and adolescents to deliver nicotine. E-cigarettes are thought to be a less harmful and more socially acceptable alternative to tobacco smoking; however, their long-term effects on health, including oral health, are currently unknown. **Methods**: A literature search for relevant papers indexed in the literature from 2016 to 2023 was conducted using the PubMed, Scopus, and Google Scholar databases. In our paper, we included clinical trials and both in vivo and in vitro research concerning the impact of e-cigarettes on oral health. **Results**: E-cigarettes impact the oral cavity, which is directly exposed to inhaled chemicals present in e-cigarette aerosols. The use of e-cigarettes has been linked to teeth discoloration and dental caries, promoting the development of periodontal diseases and causing oral mucosa lesions, including oral cancer. E-cigarette aerosols might also negatively affect the oral microbiome by suppressing the growth of commensal bacteria and increasing the population of bacteria responsible for developing numerous oral disorders. E-cigarettes also impact saliva composition and its properties, including reducing saliva’s antibacterial and antioxidant properties, which may subsequently lead to the promotion of oral diseases. **Conclusions**: The outcomes suggest that e-cigarette usage may cause the development of oral diseases, however further longitudinal studies of a larger and homogenous group of e-cigarette users are required.

## 1. Introduction

According to the World Health Organization, tobacco smoking is one of the most severe health risks in the world to both general health and the condition of the oral cavity [1,2]. Tobacco smoking is the major risk factor for precancerous conditions of the oral mucosa, such as leukoplakia, which predisposes to the development of oral mucosa lesions and periodontal diseases [3,4]. Tobacco smoking disrupts the microbiome of the oral cavity by creating an oxygen deficiency, leading to the promotion of dental plaque and interfering with neutrophil function [5,6,7]. Vasoconstriction in smokers is caused by the heat generated during smoking and the toxicants present in cigarette smoke, including carbon monoxide, hydrogen cyanide, and nicotine [8]. A severe negative impact of tobacco smoking on periodontal tissues is caused by high toxicity, impaired blood circulation in tissues, oxygen deficiency, and advancement of pathogenic bacteria growth [9]. An impairment in tissue healing, including regenerative mechanisms of bone and soft tissue among tobacco smokers, was also observed [8,10]. For these reasons, smoking cessation becomes an essential element of preventive and therapeutic interventions. Nicotine replacement therapy provides tobacco smokers with an alternative source of nicotine to satisfy their cravings. Novel non-therapeutic products such as electronic cigarettes (e-cigarettes) also deliver nicotine in the form of inhaled aerosol [11,12,13]. E-cigarettes are also used by young adults and adolescents who do not have previous experience with tobacco smoking [12,13,14,15]. Moreover, the use of e-cigarettes (commonly referred to as ‘vaping’) by users previously naïve to nicotine might present higher addictive potential than traditional cigarettes [16].

Nicotine solutions (e-liquids) used in e-cigarettes consist of propylene glycol, glycerol, nicotine, and flavoring additives. Both propylene glycol and glycerol are harmless compounds approved in the food, pharmaceutical, and cosmetic industries [17,18,19]; however, when heated to high temperatures, they may produce toxicants such as formaldehyde, which is a highly irritating and carcinogenic substance also present in tobacco smoke [20,21,22]. The main advantage of e-cigarettes is the elimination of numerous carcinogenic substances contained in tobacco smoke [21,23]. Although e-cigarettes do not produce numerous toxicants associated with cigarette smoke, such as carbon monoxide, there are multiple detrimental substances, such as nitrosamines, heavy metals, and silicate particles present in e-cigarette aerosol [24]. There are concerns about the potential negative respiratory consequences of inhaling various flavoring substances used in e-liquids. Flavoring chemicals that create menthol, tobacco, fruit, and dessert flavors are added to attract consumers to e-cigarettes. Many of those flavorings are approved for use in the food and cosmetics industries, but their effects on the respiratory systems still need to be examined [20,25,26,27,28].

An increase in the popularity of e-cigarettes has been observed in many countries. E-cigarettes were initially introduced as a less harmful and more socially acceptable alternative to tobacco smoking; however, nowadays, the safety of e-cigarette use is still unknown [23,25,29,30,31]. E-cigarette users report several side effects, including cough, dry throat, conjunctival irritation, and increased heart rate. A negative impact on the respiratory system has also been observed and reported [20,23,25,32,33,34]. E-cigarette usage promotes an inflammatory response in the respiratory system’s epithelial cells and increases pro-inflammatory interleukins 6 and 8. The higher inflammation and susceptibility to infections of the upper respiratory system among e-cigarette users might be similar to tobacco smokers [35]. Since the oral cavity is directly exposed to inhaled aerosols from e-cigarettes, similar to the respiratory system, it is expected that e-cigarettes will also impact oral health [36].

This review aims to comprehensively present the current knowledge of the impact of e-cigarette usage on oral health, including oral mucosa lesions and side effects, microbiological changes, and saliva composition and properties. Although there are several reviews regarding the impact of e-cigarettes on the oral cavity [37,38,39,40,41], those papers do not include all aspects presented in this review and some novel relevant research.

## 2. Materials and Methods

A literature search for relevant papers indexed in the literature from 2016 to 2023 was conducted using the PubMed, Scopus, and Google Scholar databases. In our paper, we included clinical trials and both in vivo and in vitro research concerning the impact of e-cigarettes on oral health. Keywords included: “e-cigarettes”, “oral health”, “oral mucosa”, “periodontitis”, “oral microbiome”, “oral candidosis”, and “saliva”. We also screened the references of the systematic reviews and meta-analyses to identify additional, original studies that were not found in our prior search. 

## 3. The Impact of E-Cigarettes on Dental and Periodontal Health

The most observed adverse events of e-cigarettes in the oral cavity were associated with irritation after inhaling aerosols emitted from those devices. Users commonly reported cough and mouth or throat irritation, which appear among almost 40% of users [42,43]; however, those adverse effects might be caused by hyperventilation, which was associated with longer puffing time on e-cigarettes compared with tobacco cigarettes [44]. Regular use of e-cigarettes might also be related to poor oral hygiene, which might be caused by a reduced amount of saliva, leading to dry mouth and plaque accumulation [45].

E-cigarettes may also pose an impact on teeth. The interaction of substances present in e-cigarette aerosol with tooth enamel could cause dental caries. Glycerin and flavoring agents might increase microbial adhesion to the tooth surface and promote biofilm formation, leading to demineralization and caries development [46]. According to Rouabhia et al., e-cigarettes increased the growth and adherence of *Streptococcus mutans*, bacteria strictly related to developing dental caries [47]. A significant biofilm growth was also observed in e-cigarette users [47]. According to Ismail et al., dental caries were significantly more advanced in a group of e-cigarette users compared with non-smokers over six months [48]. E-cigarette use was also associated with poor oral health, which was estimated by the number of removed permanent teeth. Dental caries and periodontal diseases remain the most important contributions to tooth loss [49]. Calcium, iron, and copper found in e-cigarette aerosol are involved in the mineralization and demineralization of enamel. Sweet-flavored e-liquids might promote the adherence of carcinogenic bacteria to the tooth surface and might be the reason for decreased enamel hardness after exposition to flavored e-liquids [50]. The discoloration of teeth, dental prostheses, and fillings was observed in vitro and presumably caused by flavoring substances present in e-liquids [51]. Vohra et al. observed that the discoloration of tooth restoration materials was similar to that associated with tobacco smoking [52]. The most visible discoloration effect was observed when enamel was exposed to menthol- and tobacco-flavored e-liquids [51].

E-cigarettes also pose an impact on periodontal health. The first symptom of periodontal disease is gum bleeding. The decreased gingival bleeding on probing remains an effect of tobacco smoking and leads to later diagnosis of periodontal diseases, worse prognosis, and poorer treatment results. Tobacco smokers experience greater severity of periodontal diseases, including increased pocket depth (PD), clinical attachment loss (CAL), and gingival recession. E-cigarettes also may present a suppressive effect on gums bleeding, however less intense than traditional cigarettes [53,54,55,56]. The marginal bone loss and CAL in individuals who smoked tobacco and used e-cigarettes were significantly higher than among non-smokers [57]. E-cigarette users more often reported current gum diseases or history of gum disease treatment and are more prone to develop periodontal disease than non-smokers [58]. Higher CAL, PD, and marginal bone loss were observed in e-cigarette users and cigarette smokers than among non-smokers [59]. A higher rate of severe periodontal disease occurrence among both e-cigarette users and cigarette smokers was observed; however, a unique CAL increase appeared among e-cigarette users [60]; therefore, Mokeem et al. did not observe significant differences in CAL, PD, and marginal bone level between e-cigarette users compared with non-smokers [61].

E-cigarette usage poses a negative impact on dental and periodontal health, including more advanced dental caries and periodontal diseases. The impact of e-cigarette usage on both dental and periodontal health is presented in Table 1.

## 4. The Impact of E-Cigarette Usage on Oral Mucosa Lesions

Tobacco smoking is associated with the development of various oral mucosa lesions, including leukoplakia, nicotinic stomatitis, necrotizing gingivitis, black hairy tongue, melanosis, epithelial dysplasia, and squamous-cell carcinoma. Oral lesions are also linked to e-cigarette usage [38].

According to a prospective case-control study by Bardellini et al. on a group of 90 participants (45 e-cigarette users and 45 former smokers), oral mucosa lesions were more often observed among e-cigarette users than former smokers. Oral mucosa melanosis, nicotine stomatitis, hairy tongue, and hyperplastic candidiasis were the most common oral mucosa disorders in e-cigarette users; however, some cases of median rhomboid glossitis, erythematous candidiasis, and leukoplakia were also observed [62]. It might be assumed that not only the exposure to nicotine but also to some of the toxic compounds present in e-cigarette aerosol might pose an impact on nicotine stomatitis in e-cigarette users [24,27,62]. Candida’s ability to invade superficial layers of the epithelium is optimal at acidic pH, and hyperplastic candidiasis is commonly observed in cigarette smokers [63]; however, in e-cigarette users, it might be related to a pH alteration induced by the chemical compounds in e-cigarette vapor [62]. The abnormal coating on the dorsal surface of the tongue leading to the clinically observed hairy tongue can be correlated with pH changes, mucosal dryness, and high intraoral temperatures among e-cigarette users [62].

Oral lichenoid reaction to e-cigarette use was reported by Bartram et al. In this case report, a white reticular patterned striae on the oral mucosa and lower lip was observed, and the histopathology revealed hyperkeratosis with lichenoid inflammation [64].

In a case report by Farinha et al., a hairy tongue related to e-cigarette usage was observed. The patient developed an asymptomatic black discoloration of the tongue atria after two weeks of e-cigarette use. The discontinuation of e-cigarette use resulted in spontaneous resolution of tongue discoloration; however, the discoloration re-emerged when the patient began to use the e-cigarettes again [65].

E-cigarette usage may also lead to the development of side effects on oral mucosa, such as nicotine stomatitis, hairy tongue, black tongue, altered taste perception, and burns. Those side effects might be associated with the type of used flavors, especially menthol and cinnamon flavors; however, oral mucosal side effects of e-cigarette usage tend to be less stringent than those related to tobacco smoking [66].

Baweja et al. observed side effects of oral cavities associated with e-cigarettes, including dry mouth and reduced saliva secretion, chronic bad breath, and chapped lips. Non-smokers who began using e-cigarettes experienced more severe effects on oral mucosa than former tobacco smokers [67].

Long-term e-cigarette usage might also lead to oral carcinoma development, which was presented in a case report by Nguyen et al. [68]. These two cases concerned generally healthy patients with no history of tobacco smoking or use of other tobacco products. Both patients reported everyday use of e-cigarettes for the past 13 years. In Case 1, the patient reported unintended weight loss, dysphagia, xerostomia, and paresthesia of the tongue. Clinically, Some exophytic masses with surrounding hyperkeratotic areas resembling features of lichen planus were observed. The histopathology revealed the basaloid squamous cell carcinoma of the anterior aspect of the tongue. In Case 2, the patient had a non-healing lower lip ulceration that appeared nine months before. The subjective symptoms included swallowing difficulty and xerostomia. No pain and history of trauma was reported. The clinical examination revealed a 1 cm ulcerative lesion on the vermilion of the lower lip. No other extra- and intraoral abnormalities were observed. The histopathological examination led to a diagnosis of basaloid squamous cell carcinoma [68].

E-cigarette usage might be associated with dry mouth and chronic bad breath and lead to the development of oral mucosa lesions such as nicotine stomatitis, melanosis, and hairy tongue; therefore, some cases of oral carcinoma associated with long-term e-cigarette usage were observed. Oral mucosa lesions related to e-cigarette usage are presented in Table 2.

## 5. The Impact of E-Cigarettes on the Oral Microbiome

The oral cavity remains a highly heterogeneous ecosystem inhabited by more than 700 bacterial species. This physiological bacterial flora provides oral homeostasis and a proper function of the oral cavity and the whole organism. Any disorders resulting from metabolic and environmental factors impair homeostasis and may impact oral and general disease development. The dysbiosis in oral microflora leads to dental caries and halitosis or contributes to diabetes, cardiovascular diseases, and cancers [69,70]. It has also been proven that tobacco usage strongly impacts periodontal health and remains a significant risk factor for periodontitis development [71]. The oral cavity is highly exposed to toxic components and nicotine present in e-liquids, and the interaction of those chemical substances with the microbiome may result in higher susceptibility to infections [72].

According to Pushalkar et al., e-cigarette users were characterized by a greater abundance of Gram-negative bacteria *Porphyromonas* and *Veillonella*, especially *V. atypica* and *V. rogosae*, compared with tobacco smokers and non-smokers [68]. *Veillonella* spp. is a comical bacteria that mainly exists on the dorsal and lateral surfaces of the young and is present in saliva. The correlation between *Veillonella* spp. in tobacco smoking patients with periodontitis remains unclear; however, it has been suggested that *V. atypica* may convert nitrate to nitrite, which can be further transformed into nitrosamines and nitric oxide. Nitrosamines are toxic and potentially carcinogenic chemical compounds related to tobacco smoking, whereas nitric oxide has a pro-inflammatory effect [73,74,75]. *Porphyromonas gingivalis* is an anaerobic Gram-negative bacterium classified as red Socransky’s complex and a significant risk factor for periodontitis development. The immunological mechanism interferes with neutrophil recruitment by deactivating IL-8 production, limiting E-selectin production, degradation of essential complement proteins such as C3 and C5 induced by gingipain, and impeding the complement cascade [76].

In research conducted by Catala-Valentin et al., it was observed that e-cigarette aerosols limited the growth of *S. sanguinis* and *S. gordonii* but did not impact the growth of *S. mutans*. E-cigarette aerosols also significantly increased biofilm formation by *S. mutans* but did not affect the biofilm formation by *S. sanguinis* and *S. gordonii* [77]. Although the oral microbiome is a highly heterogeneous ecosystem, the most prevalent bacteria are *Streptococcus* spp. *S. sanguinis* and *S. gordonii* are commensals dominating the phase of homeostasis. *S. mutans* is an opportunistic bacterium typically observed in the oral cavity as part of a mature dental biofilm; due to environmental changes, it may dominate over other species, leading to disorders in oral homeostasis. *S. mutans* is associated with dental caries and severe periodontitis development. [78]. The results suggest that e-cigarette aerosols might negatively affect oral bacterial homeostasis by the growth limitation of commensal bacteria *S. sanguinis* and *S. gordonii* while promoting the biofilm formation of the opportunistic bacteria *S. mutans* [77].

Therefore, Nelson et al. [79] and Cuadra et al. [80] did not observe a significant impact of e-cigarette usage on the growth of *Streptococcus* commensal bacteria, including *S. gordonii*, *S. mitis*, and *S. oralis*. In research conducted by Nelson et al., the exposure to nicotine, e-liquids with and without nicotine, and e-cigarette aerosol with and without nicotine did not significantly change the growth of *S. gordonii*, *S. mitis*, and *S. oralis*. In contrast, exposure to cigarette smoke caused a dose-dependent decrease in bacterial growth [79]. In research conducted by Cuadra et al., no adverse effect on the growth of commensal *S. gordonii, S. intermedius, S. mitis*, and *S. oralis* bacteria exposed to flavorless e-cigarette aerosol with and without nicotine was observed. In contrast, the exposure to cigarette smoke severely impaired bacteria growth [80].

According to Chopyk et al., e-cigarette users presented a significant increase in *Veillonella* and *Haemophilus* bacteria in the oral cavity compared with non-smokers. A significantly different oral microbiome composition that was observed in e-cigarette users may suggest that e-cigarette use results in dysbiosis of the oral environment, which might be associated with systematic disease development [81]. *Veilonella* spp. and *Haemophilus* spp. are bacteria often observed in the oral cavity and upper respiratory tract; therefore, *Haemophilus influenza* is a contributor to lung diseases associated with tobacco smoking and is commonly present in the lower airways of patients with chronic obstructive pulmonary disease [82]. *H. influenza* isolates, when exposed in vitro to e-cigarette aerosol, increased the degree of biofilm formation and provoked a more significant inflammatory response in human airway epithelial cells [83].

Ganesan et al. suggest that the most critical effect of e-cigarette usage on the oral microbiome is the modification of biofilm architecture. E-cigarette use was also related to higher levels of Gram-negative facultative bacteria, whereas tobacco smoking was associated with selective enrichment in Gram-negative anaerobic bacteria [84].

It has been established that tobacco smoke is a significant environmental factor affecting oral microorganisms, including *C. albicans*, and an etiological factor of oral candidiasis [85]. In research conducted by Haghighi et al., both traditional cigarettes and e-cigarettes with nicotine presented inhibitory effects against *C. albicans* at nicotine levels that were about an order of magnitude lower than for pure nicotine; however, e-cigarettes without nicotine did not impact the growth of *C. albicans*, which may suggest that *C. albicans* are more susceptible to nicotine than other components present in e-cigarette liquids [86].

According to Mokeem et al., colonization by *Candida* spp. is significantly more frequently observed among both tobacco smokers and e-cigarette users compared with non-smokers; therefore, *Candida* spp. was isolated more often in tobacco smokers than e-cigarette users [61].

In research conducted on a group of 125 patients, a significant increase in Gram-negative rods among e-cigarette users in comparison to non-smokers was observed [87]. Gram-negative rods might temporarily colonize the upper respiratory tract; however, their occurrence in the oral microflora may pose a potential risk for pulmonary infections [88]; however, no differences in *C. albicans* occurrence among e-cigarette users compared with non-smokers were observed [87].

Therefore, in research conducted by Stewart et al., no essential changes in oral bacteria diversity among electronic cigarette users compared with non-smokers were observed [89].

Xu et al. observed the enrichment of *Treponema* and *Fusobacterium* bacteria in the saliva of e-cigarette users. The alteration of saliva bacteria composition among e-cigarette users and tobacco smokers was similar. It may increase periodontitis-related bacteria, such as *Porphyromonas gingivalis* and *Fusobacterium nucleatum* [90].

In research conducted by Wang et al., there were differences in the structure and composition of the oral microbic between e-cigarette users and tobacco smokers. Among e-cigarette users, the most commonly present bacteria were *Campylobacter, Veillonella, Prevotellaceae*, and *Prevotella,* whereas in tobacco smokers dominated *Porphyromonas, Fusobacterium,* and *Haemophilus* taxa. The abundance of *Neisseria* in the e-cigarette group was significantly lower than that among tobacco smokers and non-smokers [91]. Smoking impacts the oral microbiome by creating anaerobic conditions that favor anaerobic microorganisms, such as *Veillonella* and *Fusobacterium*, and suppresses aerobic organisms, such as *Neisseria* [92]. It was observed that smoking cessation could lead to the re-establishment of the oral microbiome to that of non-smokers [91].

According to Park et al., a significant increase in α-diversity in the saliva and subgingival sites for e-cigarette users compared with non-smokers was observed. This leads to the conclusion that exposure to e-cigarette vapor may cause an increase in microbial diversity and result in microbial dysbiosis, leading to periodontal diseases [93].

The majority of research observed that e-cigarette aerosols pose an impact on the oral microbiome by suppressing the growth of commensal bacteria and promoting the growth of bacteria such as *Porphyromonas* and *Fusobacterium* that participate in the development of oral disorders such as periodontitis. Some research also observed an increase in the colonization of *C. albicans* among e-cigarette users; therefore, further research is required to assess the longitudinal impact of e-cigarettes on the oral microbiome. The observed effects of e-cigarettes on the oral microbiome are presented in Table 3.

## 6. The Impact of E-Cigarettes on the Composition and Properties of Saliva

Saliva is a secretion constantly produced by salivary glands that ensure homeostasis maintenance in the oral cavity [94]. Saliva primarily consists of water (99%); however, the physiochemical properties of saliva are determined by the presence of numerous inorganic and organic substances that nourish and protect the surrounding tissues. Saliva composition might be affected by various factors, including tobacco smoking [95]. It has also been proven that chemical compounds in e-cigarette vapor dissolve in saliva and impact its biochemical composition and function.

Lactate dehydrogenase (LDH) is a cytoplasmic enzyme in the human body’s cells that might be released, raising its level in serum and saliva, related to oxidative stress. The extracellular leakage of this enzyme might indicate cell damage or death [96]. Salivary LDH concentrations might be considered an indicator of the effect of mucosal damage leading to the loss of integrity of the oral mucosa [97]. In recently conducted research, e-cigarette users presented the highest salivary LDH levels compared with cigarette smokers and non-smokers, which indicates higher cell death and oral epithelial cell breakdown among e-cigarette users [98].

In recent research, the uric acid, hypoxanthine, xanthine, TAOS (total antioxidant status), and TEAC (Trolox equivalent antioxidant capacity) were determined in the saliva samples of e-cigarette users. The values of TAOS and TEAC were significantly lower than in non-smokers. Both e-cigarettes and traditional cigarettes have a military impact on saliva antioxidant capacity [99]. The impaired antioxidant function of saliva can impact the formation of free radicals and reactive oxygen species (ROS), which play a significant role in the progression of periodontitis and the destruction of oral mucosa tissue by promoting inflammatory reactions [100].

E-cigarette usage also has an impact on the antibacterial properties of saliva. The values of IgA and lysosome in saliva in a group of e-cigarette users were lower in comparison to non-smokers, similarly to cigarette smokers; therefore, the values of lactoferrin were higher than in non-smokers, in contrast to cigarette smokers [101]. IgA values in saliva are relatively low; however, they increase among patients with periodontitis, presenting a defense mechanism against antigens of bacterial biofilm. Lowering the value of IgA in saliva may result in impairment of specific immune responses and lead to more severe periodontitis [102]. Lysosome is an enzyme presenting antibacterial properties by causing lysis of bacterial cell walls; however, it also has antiviral and antifungal activity [103]. Lactoferrin is an iron-binding glycoprotein with immunomodulatory and anti-inflammatory properties, and its increased values indicate the currently occurring inflammatory processes [104].

According to Ye et al., there were no significant differences in saliva values inflammatory markers such as prostaglandin E2 (PGE2) and interleukin 1β (IL-1β) levels in e-cigarette users compared with non-smokers and lower to cigarette smokers. The level of MPO (myeloperoxidase), which is an oxidative stress marker, was significantly reduced in e-cigarette users compared with non-smokers and cigarette smokers. These results suggest that long-term e-cigarette usage might be associated with the development of chronic systemic and oral disease; however, less than cigarette smoking [105].

In research conducted by Singh et al. in the saliva of e-cigarette users, a significant increase in IL-1β compared with non-smokers was observed. An increase in IL-6, resolvin D2, and a decrease in resolvin D1 and prostaglandin E2 was observed in e-cigarette users compared with non-smokers was also observed; however, there was no statistical significance [106].

Pushalkar et al. observed that interleukins IL-6 and IL-1β in the saliva of e-cigarette users were highly elevated in comparison to non-smokers [73]. IL-6, IL-1β, and TNF-α are mediators in developing dental and periodontal diseases, including stimulation of tissue degradation due to increased matrix metalloproteinases. High levels of IL-6 are observed in patients with periodontitis. Increased inflammation in the oral cavity remains a risk factor for the development of periodontal diseases [107]. Research conducted by Verma et al. also concerned the impact of e-cigarette usage on cytokines levels. It revealed an increased salivary level of pro-inflammatory cytokines, including TNF-α and IL-1 β, with decreased levels of anti-inflammatory cytokine IL-1RA among e-cigarette users compared with non-smokers [108]. In research conducted by Alqahtani et al., an increased level of pro-inflammatory cytokines IL-1β and TNF-α in the saliva of e-cigarette users compared with non-smokers were observed, as well as increased levels of prostaglandins and leukotrienes suggesting an increased arachidonic acid metabolism in e-cigarette users [109].

Faridoun et al. also observed an increased level of pro-inflammatory cytokines IL-1β and TNF-α in the saliva of e-cigarette users compared with non-smokers. In contrast, there were no differences in levels of IL-6 and IL-8 between those two groups [110].

However, Mokeem et al. observed that levels of IL-1β and IL-6 in e-cigarette users were similar to non-smokers and lower than among cigarette smokers [54]. Increases in the levels of pro-inflammatory cytokines IL-15 and IL-18 were observed by Ali et al. among e-cigarette users and cigarette smokers. IL-15 and IL-18 levels were higher in a group of non-smokers with periodontitis than in non-smokers without periodontitis [111].

Salivary pH values were higher in a group of e-cigarette users compared with cigarette smokers and non-smokers [73]. The saliva pH should range from 6.2 to 7.6. Maintaining proper saliva pH depends on the saliva’s buffering activity and the constant salivary flow. It was observed that saliva pH was lower in patients with periodontitis [112]. Saliva pH value could be affected by tobacco usage [113,114].

Another research observed that among e-cigarette users, the value of calcium in saliva was higher than in the group of non-smokers; however, no significant differences in the pH value or the concentration of total protein and phosphates were observed [115]. An increased concentration of inorganic components in saliva, such as calcium, might result in higher mineralization of dental plaque, which remains a list factor for periodontal diseases and dental caries [116,117].

The majority of research observed that e-cigarettes pose an impact on saliva composition and properties, including a reduction in saliva’s antibacterial and antioxidant abilities. Levels of proinflammatory cytokines, in general, were higher among e-cigarette users compared with non-smokers, which may lead to the promotion of oral diseases; however, further research is required to assess the longitudinal impact of e-cigarettes on saliva. The observed effects of e-cigarettes on saliva composition are presented in Table 4.

## 7. Conclusions

This review summarizes the current research on the impact of e-cigarettes on oral health. The outcomes suggest that e-cigarette usage may cause the development of oral diseases, including teeth discoloration and dental caries, periodontal diseases, and oral mucosa lesions that may lead to oral cancer. E-cigarette aerosols also negatively affect the oral microbiome by suppressing commensal bacteria growth and increasing bacteria participating in developing oral disorders such as periodontitis. E-cigarettes also pose an impact on saliva composition and properties, including a reduction in saliva’s antibacterial and antioxidant abilities, which may lead to the promotion of oral diseases; however, the results presented in those studies differ from one another, and further longitudinal studies of a larger and homogenous group of e-cigarette users are required.

## 8. Limitations of the Study

A significant limitation of this review is that the studies were conducted on a heterogeneous group of e-cigarette users. The nicotine concentration, flavoring substances, duration of e-cigarette usage, and patient’s inclusion and exclusion criteria differ from one another; therefore, not all studies were performed strictly to evaluate the oral health effects.

## Figures and Tables

**Table 1:** The impact of e-cigarettes on dental and periodontal health.

Authors	The Impact of E-Cigarettes on Dental and Periodontal Health	Type of Study
Rouabhia et al. [47]	Increased growth and adherence of *S. mutans*	in vitro
Ismail et al. [48]	More advanced dental caries in e-cigarette users	prospective
Huilgol et al. [49]	E-cigarette usage is associated with poor oral health	cross-sectional
BinShabaib [57]	Higher CAL and marginal bone loss in e-cigarette users and cigarette smokers	cross-sectional
Vora [58]	Gum disease more often reported by e-cigarette users than non-smokers	case controlled
Ibraheem et al. [59]	Higher CAL, PD, and marginal bone level in e-cigarette users and cigarette smokers than non-smokers	case controlled
Xu et al. [60]	Increased CAL in e-cigarette users, higher rate of severe periodontal disease in cigarette smokers and e-cigarette users	longitudinal
Mokeem et al. [61]	No differences in CAL, PD, and marginal bone level among e-cigarette users compared with non-smokers	cross-sectional

CAL—clinical attachment loss; PD—increased pocket depth.

**Table 2:** Oral mucosa lesions in e-cigarette users.

Authors	Oral Mucosa Lesions in E-Cigarette Users	Type of Study
Bardellini et al. [62]	Oral mucosa melanosis, nicotine stomatitis, hairy tongue and hyperplastic candidiasis	prospective case-control
Bartram et al. [64]	Oral lichenoid reaction to e-cigarette use	case report
Farinha et al. [65]	Hairy tongue related to e-cigarette use	case report
Li et al. [66]	Nicotine stomatitis, hairy tongue, black tongue, and burns	social media
Baweja et al. [67]	Dry mouth, bad breath, and chapped lips	cross-sectional
Nguyen et al. [68]	Oral carcinoma	case report

**Table 3:** The impact of e-cigarette usage on the oral microbiome.

Authors	The Impact of E-Cigarette Usage on the Oral Microbiome	Type of Study
Pushalkar et al. [73]	Greater abundance of Gram-negative bacteria *Porphyromonas* and *Veillonella* compared with tobacco smokers and non-smokers	cross-sectional and in vitro
Catala-Valentin et al. [77]	Growth suppression of *S. sanguinis* and *S. gordonii*; no impact on the growth of *S. mutans; an* increase in biofilm formation by *S. mutans*	in vitro
Nelson et al. [79]	No impact on the growth of *Streptococcus* commensal bacteria compared with cigarette smoke	in vitro
Cuadra et al. [80]	No impact on the growth of *Streptococcus* commensal bacteria compared with cigarette smoke	in vitro
Chopyk et al. [81]	A significant increase in *Veillonella* and *Haemophilus* in e-cigarette users compared with non-smokers	cross-sectional
Ganesan et al. [84]	A modification of biofilm architecture and higher levels of Gram-negative facultative bacteria in e-cigarette users compared with cigarette smoke	cross-sectional
Haghighi et al. [86]	Inhibitory effects of e-cigarettes with nicotine against *C. albicans;* e-cigarettes without nicotine did not impact the growth of *C. albicans*	in vitro
Cichońska et al. [87]	A significant increase in Gram-negative rods in e-cigarette users compared with non-smokers; no differences in *C. albicans* occurrence	cross-sectional
Mokeem et al. [61]	More frequent colonization of *C. albicans* in e-cigarette users compared with non-smokers	cross-sectional
Stewart et al. [89]	No changes in oral microbiota among e-cigarette users compared with non-smokers	cross-sectional
Xu et al. [90]	Increase in *Treponema* and *Fusobacterium* bacteria in the saliva of e-cigarette users compared with cigarette smoke	cross-sectional
Wang et al. [91]	Increase in *Campylobacter*, *Veillonella*, *Prevotellaceae*, and *Prevotella;* decrease jn *Neisseria* in e-cigarette users compared with tobacco smokers and non-smokers	cross-sectional
Park et al. [93]	Increase in α-diversity in e-cigarette users compared with non-smokers	cross-sectional and in vitro

**Table 4:** The impact of e-cigarette usage on saliva composition and properties.

	The Impact of E-Cigarette Usage on Saliva	Type of Study
Pushalkar et al. [73]	Higher salivary levels of IL-6 and IL-1β in e-cigarette users compared with non-smokers	cross-sectional and in vitro
Pandarathodiyil et al. [98]	Higher salivary LDH levels and pH values in e-cigarette users compared with non-smokers and cigarette smokers	cross-sectional
Cichońska et al. [99]	Lower TAOS and TEAC values compared with non-smokers	cross-sectional
Cichońska et al. [101]	Lower values of IgA and lysosome and higher values of lactoferrin in e-cigarette users compared with non-smokers	cross-sectional
Ye et al. [105]	Reduced level of MPO, no differences in PGE2 and IL-1*β* in e-cigarette users compared w non-smokers	cross-sectional
Singh et al. [106]	Increased value of IL-1β in e-cigarette users compared with non-smokers	cross-sectional
Verma et al. [108]	Higher levels of TNF-α and IL-1β, lower level of IL-1RA in e-cigarette users compared with non-smokers	cross-sectional
Alqahtani et al. [109]	Higher levels of TNF-α, IL-1β, prostaglandins, and leukotrienes compared with non-smokers	cross-sectional
Faridoun et al. [110]	Higher level of IL-1β and TNF-α in saliva; no differences in levels of IL-6 and IL-8 compared with non-smokers	cross-sectional
Mokeem et al. [54]	Levels of IL-1β and IL-6 in e-cigarette users are similar to non-smokers and lower than in cigarette smokers	cross-sectional
Ali et al. [111]	Higher levels of IL-15 and IL-18 in saliva in e-cigarette users and cigarette smokers	case controlled
Cichońska et al. [115]	Higher values of calcium in e-cigarette users compared with non-smokers, no differences in pH value, total protein, and phosphates	cross-sectional

## Data Availability

No new data were created or analyzed in this study. Data sharing is not applicable to this article.
